# Droplet digital PCR as alternative to microbiological culture for *Mycobacterium tuberculosis* complex detection in bovine lymph node tissue samples

**DOI:** 10.3389/fcimb.2024.1349999

**Published:** 2024-02-26

**Authors:** José María Sánchez-Carvajal, Eduardo Vera-Salmoral, Belén Huerta, Ángela Galán-Relaño, Inés Ruedas-Torres, Fernanda Larenas-Muñoz, Inmaculada Luque, Librado Carrasco, Jaime Gómez-Laguna

**Affiliations:** ^1^ Department of Anatomy and Comparative Pathology and Toxicology, Pathology and Immunology Group (UCO-PIG), Unidad de Investigación Competitiva (UIC) Zoonosis y Enfermedades Emergentes ENZOEM, University of Córdoba, Córdoba, Spain; ^2^ Department of Animal Health, Unidad de Investigación Competitiva (UIC) Zoonosis y Enfermedades Emergentes (ENZOEM), University of Córdoba, University of Córdoba, Córdoba, Spain; ^3^ UK Health Security Agency (UKHSA), Salisbury, United Kingdom

**Keywords:** droplet digital PCR, bovine tuberculosis, *Mycobacterium tuberculosis* complex, molecular diagnosis, IS6110, lymph node, ddPCR

## Abstract

**Introduction:**

Bovine tuberculosis (bTB) caused by *Mycobacterium tuberculosis* complex (MTC) remains a significant concern for public health. Direct real-time PCR and droplet digital PCR (ddPCR) are proposed as alternative tools to enhance diagnostic precision and efficiency. This study aims to assess the diagnostic performance of a ddPCR assay targeting IS*6110* for the detection of MTC DNA in both microbiological culture and fresh lymph node (LN) tissue samples obtained from cattle, in comparison with the established reference standard, the microbiological culture followed by real-time PCR.

**Methods:**

The fresh LNs (N=100) were collected each from a different cattle carcass at the slaughterhouse. The limit of detection of ddPCR-IS*6110* was set to 101 copies per 20 μl reaction.

**Results:**

DdPCR-IS*6110* detected 44 out of 49 reference-standard positive samples and yielded negative results in 47 out of 51 reference-standard negative samples, resulting in adjusted sensitivity (Se) and specificity (Sp) of 90.76% [95% confidence interval (CI): 82.58 - 98.96%)], and 100% (95% CI: 100%) respectively. The estimated adjusted false negative rate (FNR) was 9.23% (95% CI: 1.04 - 17.42%) and the false positive rate (FPR) was 0% (95% CI: 0%). When directly applied from fresh bovine LN tissues, ddPCR-IS6110 identified 47 out of 49 reference-standard positive samples as ddPCR-IS6110-positive and 42 out of 51 reference-standard negative samples as ddPCR-IS*6110*-negative, resulting in adjusted Se and Sp values of 94.80% [95% (CI): 88.52 - 100%] and 100% (95% CI: 100%), respectively. The adjusted FNR was 5.20% (95% CI: 0 - 11.50%) and the FPR was 0% (95% CI: 0%). Noteworthy, ddPCR-IS*6110* disclosed as positive 9 samples negative to reference-standard.

**Discussion:**

DdPCR-IS*6110* proved to be a rapid, highly sensitive, and specific diagnostic tool as an alternative to reference-standard method.

## 1 Introduction

Bovine tuberculosis (bTB) is caused by bacteria belonging to *Mycobacterium tuberculosis* complex (MTC), mainly *M. bovis* and *M. caprae* (Aranaz et al., 2004; Domingo et al., 2014). bTB is still a zoonoses of major concern for public health, especially in developing countries (Dean et al., 2018). In the European Union (EU), even though the eradication is the main goal, this disease is still present in dairy and beef herds (MAPA, 2022). Current European approved surveillance systems are based on detection of a specific cellular immune reaction by single or comparative intradermal tuberculin testing (SIT or SCIT) and interferon gamma release assays (IGRA), followed by compulsory slaughter of reactor animals as well as post-mortem confirmation. Furthermore, this program includes abattoir surveillance for undetected bTB-infected animals, regular retesting and culling of infected animals and restrictions on the movement of livestock to prevent introduction of infected animals (EU, 2023).

These approaches have simplified the diagnosis of MTC-infected cattle at the early stage of the disease (de la Rua-Domenech et al., 2006), therefore, animals with clinical signs or gross tuberculosis-like lesions (TBL) are lack or rarely found at the slaughterhouse (de la Rua-Domenech et al., 2006; Ramos et al., 2015; Pozo et al., 2021). This success of surveillance systems has challenged the traditional examination of atypical or enlarged lymph nodes (LNs) or parenchymatous organs with gross TBLs, and/or culture of MTC in primary isolation medium followed by real-time PCR. In this scenario, several factors could play a role in hindering the eradication of bTB. One of the most relevant drawbacks is the poor diagnostic performance reported for the current diagnostic tools. As a consequence, truly infected animals are misclassified as bTB-free, which contribute to maintaining the chain of infection on the farm, but also in sharing pasture areas.

In order to make the diagnostic and confirmation procedure for bTB more reliable and swifter, several diagnostic tools have been proposed as alternatives to the reference standard (microbiological culture followed by confirmation by real-time PCR), which is considered an imperfect reference technique taking up to three months to obtain a confirmatory result (Courcoul et al., 2014; Lorente-Leal et al., 2021; Sánchez-Carvajal et al., 2021). Direct real-time PCR from tissue samples has been reported to be a potential first-line technique for the detection of MTC species in animal tissues worldwide (Courcoul et al., 2014; Lorente-Leal et al., 2019; Wang et al., 2019; Lorente-Leal et al., 2021; Sánchez-Carvajal et al., 2021). Nevertheless, although direct real-time PCR seems to be a simple, rapid and robust alternative to microbiological culture, PCR results could be affected mainly by the characteristics of the lesion (necrosis, calcification or fibrosis), a low mycobacterial load, the DNA isolation procedure and the presence of inhibitors (Lorente-Leal et al., 2019; Sánchez-Carvajal et al., 2021).

These limiting factors could be overcome by other molecular tools such as droplet digital PCR (ddPCR). The ddPCR is an emerging PCR assay, based on water–oil emulsion droplet technology, which have been described in several medical fields in recent years, including diagnosis of several infectious pathogens, DNA methylation determination, gene expression, and gene mutation analysis (Strain et al., 2013; Lillsunde Larsson and Helenius, 2017; Guil-Luna et al., 2023). Each sample is partitioned into approximately 20,000 droplets before being subjected to the PCR and, therefore, each droplet could contain one target molecule or none. This is a substantial advantage compared to real-time PCR, since ddPCR has been reported to be less sensitive to inhibitors due to sample partitioning (Dingle et al., 2013; Yang et al., 2014). Another key argument to bear in mind is that ddPCR is more sensitive and accurate than real-time PCR, especially in the case of low-copy acid nucleic (Devonshire et al., 2015; Yang et al., 2017; Nyaruaba et al., 2020). This technology has been already reported for the detection of *Mycobacterium tuberculosis* in human samples (Devonshire et al., 2015; Yang et al., 2017; Cao et al., 2020), or recently, for the detection of *Mycobacterium avium* subsp. *paratuberculosis* DNA in whole-blood and faecal samples from cattle (Badia-Bringué et al., 2022). However, ddPCR capability to detect MTC in fresh bovine tissue samples has not been yet evaluated. Thus, the primary aim of this research was to assess the diagnostic performance of a ddPCR assay targeting IS*6110* for the rapid and sensitive detection of MTC DNA in both microbiological culture and fresh LN tissue samples obtained from cattle, in comparison with the established reference standard.

## 2 Materials and methods

### 2.1 Samples selection and processing

This study was part of a larger project focused on developing rapid and accurate diagnostic tools in the current framework of bTB surveillance and control programs. LN tissue samples (N=100) were collected each from a different cattle carcass at the slaughterhouse from 2018 to 2019 in the context of Spanish bTB eradication program. All samples were collected during routine post-mortem veterinary examination within an official context and according with national and European regulations. No purpose killing of animals was performed for this study, so no ethical or farmer’s consent approval was required.

LNs were independently sliced to confirm the presence of visible TBLs (N=19) or no visible lesions (NVLs) (N=81) and fixed in 10% neutral-buffered formalin for the histopathological analysis. Each LN was then individually homogenized using a tissue homogenizer (Fisherbrand, Fisher Scientific, Madrid, Spain) to obtain a uniform mixture. Tissue homogenate was divided into paired samples that were used for DNA isolation and selective microbiological culture.

For histopathological evaluation, formalin fixed LNs were processed and embedded in paraffin following standard procedures. Four-micron sections were stained with haematoxylin-eosin (H&E) and Ziehl-Neelsen (ZN). Histopathological findings were classified as TBLs for those samples with a tuberculous granuloma, pyogranuloma, or scattered Langhans-type multinucleated giant cells (MNGCs), or as no histopathological lesion (NHLs), for the tissue with normal histological characteristics and no lesion compatible with TBL (Larenas-Muñoz et al., 2022). In addition, Ziehl-Neelsen (ZN) technique was performed to detect acid-fast bacilli (AFB). A sample was considered positive for ZN when one or more AFB were found in at least one high-power field magnification (HPF, 100x). The lesions were classified as paucibacillary if it was observed with 1 to 10 AFB bacilli, or pluribacillary if ≥ 11 AFB were observed per HPF (Larenas-Muñoz et al., 2022).

For the reference standard (microbiological culture followed by real-time PCR-IS*6110*), tissue homogenates were decontaminated with an equal volume of 0.75% (w/v: 1/1) hexadecyl pyridinium chloride solution in agitation for 30 min. Samples were centrifuged for 30 min at 1,500 × *g*. The pellets were collected with swabs and cultured in liquid media (MGIT™ 960, Becton Dickinson, Madrid, Spain) using an automatized BD Bacter™ MGIT™ System (Becton Dickinson) (Corner and Trajstman, 1988). DNA extraction was performed using the MagMAX Total Nucleic Acid Isolation Kit according to the manufacturer’s instructions (Thermo Fisher Scientific, Lissieu, France). DNA was eluted in 50 μl. Then, cultures were considered positive when isolates were confirmed as MTC using real-time PCR (Thierry et al., 1990). The cut-off value of real-time PCR-IS*6110* assay was set at 10 to 100 genomic equivalents, and the cut-off set at Cq ≤ 38 (10 to 100 genomic equivalents/15 μL reaction mixture) (Sánchez-Carvajal et al., 2021).

### 2.2 DNA isolation from microbiological culture and LNs

Genomic DNA isolation was conducted from tissue homogenate according to Vera-Salmoral et al., 2023 with several modifications using NucleoSpin Tissue Kit^®^ (Macherey-Nagel, Düren, Germany). In brief, 1 mL of homogenized tissue was centrifuged during 5 min at 9,000 *g*. The supernatant was discarded, and the resulting tissue pellet was added in a tube together with 250 µl of Sample Buffer T1, 150 mg of 0.5-mm glass beads and 50 mg of 0.1-mm glass beads. Then, samples were subjected to mechanical disruption (SI™ Disruptor Genie™, Scientific Industries, New York, USA) (2,850 rpm/50 Hz/20 min). After an overnight enzymatic digestion at 56°C with 30 µl proteinase K in a thermo-shaker (600 rpm/12 h), a new mechanical disruption step was conducted. Samples were centrifuged 2 min at 9,000 *g*, and pellets were again subjected to the steps described above. Then, samples were mixed with 200 µl of buffer T3, incubating the mixture for 10 min at 70°C. The lysate was transferred to a silica-based nucleic acid purification column and managed according to manufacturer’s instructions. Isolated DNA samples were stored at −20°C until used in downstream PCR assays. Positive and negative extraction controls were included.

### 2.3 Primers and probe targeting IS*6110*

Specific primers and probe were based on the homology region of the partial insertion sequence *6110* (IS*6110*), a repetitive mobile element specific for all the pathogens belonging to MTC widely used to diagnose and genotype this pathogen. The fluorogenic IS*6110*-probe was labelled with a fluorescent reporter dye [6-carboxyfluorescein (FAM)] at the 5′-end and a 3′-Black Hole Quencher 1 (BHQ1). Primers and probe used for real-time PCR and ddPCR are listed in **Table 1** (Sánchez-Carvajal et al., 2021; Lorente-Leal et al., 2021).

**Table 1:** Sequences of MTC specific primers and TaqMan probe targeting IS*6110* for real-time PCR and droplet digital PCR (ddPCR) assays.

Target	Forward primer (5′-3′)	Reverse primer (5′-3′)	Probe (5′-3′)	Amplicon (bps)
IS*6110*	GGTAGCAGACCTCACCTATGTGT	AGGCGTCGGTGACAAAGG	5’-6FAM-CACGTAGGCGAACCC-BHQ1-3’	68

### 2.4 Real-time PCR targeting IS*6110*

QuantiFast^®^ Pathogen PCR + IC Kit (QIAGEN, Hilden, Germany) was used to conduct the real-time PCR-IS*6110* evaluating each sample in duplicate in the MyiQ™2 Two-Color real-time PCR Detection System (Bio-Rad, Hercules, Ca, USA) under the following cycling conditions: 95°C for 5 min to activate the DNA polymerase followed by 42 amplification cycles that consisted of a denaturation step at 95°C for 15 s, an annealing-extension step at 60°C for 30 s. Following manufacturer’s guidelines, an exogenous inhibition heterologous control (internal amplification control, IAC) supplied with the kit was included. An inter-run calibrator with a known quantification cycle (Cq) value of 32 was introduced in each assay to self-control intra-assay. Complete inhibition of amplification was considered when IAC did not amplify and partial inhibition when it showed a Cq > 33. The analytical sensitivity or limit of detection (LOD) was estimated for the proposed primers and probes. LOD is defined as the lowest concentration in which 95% of replicates were positive, according to the Clinical and Laboratory Standard Institute guidelines. A serial 10-fold dilution series of *M. bovis* genomic DNA with known quantities ranging from 10^6^ to 10^0^ were used. The reactions were performed in triplicate for each dilution in three different assays. Thus, the LOD was determined to be ranging from 10 to 100 genomic equivalents, and the cut-off was established at Cq < 38 (Sánchez-Carvajal et al., 2021).

### 2.5 ddPCR targeting IS*6110* for MTC detection in microbiological culture and LNs

For ddPCR assay targeting IS*6110*, QX200™ ddPCR™ Supermix for probe (No dUTP) (Bio-Rad, Hercules, CA, USA) was used according to Bio-Rad ddPCR system guidelines. Each sample was evaluated in duplicate in a reaction mix with a final volume of 21 μl as follows: 10.5 μl of 1x ddPCR Supermix for probe (No dUTP), 1.7 μl of IS*6110*-forward (900 nM), 0.85 μl of IS*6110*-reverse (600 nM), 0.65 μl of IS*6110*-probe (FAM-labelled, 200 mM), 3 μl of template, and 4.3 μl of nuclease-free water. It is important to mention that several protocols for ddPCR recommend performing a restriction digestion of DNA samples outside the amplicon in order to make the template more accessible reducing sample viscosity. Nevertheless, we decided to not use restriction enzymes due to the extraction protocol herein reported got an efficient reduction of host DNA ranging from 50 – 100 ng/μl. Afterwards, the droplets were generated on the QX200 Droplet Generator (Bio-Rad, USA) using 70 μl of droplet generation oil for Probes^®^ (Bio-Rad, USA) dispatched into the bottom of the oil wells of the DG8™ Cartridge droplet generator (Bio-rad, USA) according to the manufacturer’s instructions. The droplets were carefully transferred to a specific 96-well ddPCR reaction plate (Bio-Rad, USA) using a RAININ Pipet-Lite Multi Pipette (Mettler Toledo, Columbus, Ohio, USA). After heat sealing by PX1™ PCR Plate Sealer (Bio-Rad, USA) at 180°C for 5 s, amplifications were run in the C1000 Touch thermal cycler (Bio-Rad, Hercules, CA, USA) under the following cycling conditions: 95°C for 10 min, followed by 40 cycles of 94°C for 30 s and 60°C (annealing/extension) for 1 min, and finally 98°C for 10 min. The temperature ramping rate was set at 2°C/s. Thereafter, the droplets were stored in darkness at 4°C for 12 h.

### 2.6 Limit of detection and limit of blank of ddPCR targeting IS*6110* assay

The limit of detection (LOD) was determined to be the lowest concentration of IS*6110* copies at which detection is possible (Armbruster and Pry, 2008). LOD for ddPCR-IS*6110* was determined by measuring three concentrations around LOD. Reactions were run in triplicates for each concentration (*M. bovis* genomic DNA with known quantities ranging from 10^4^ to 10^0^), and LOD was defined as the lowest concentration in which 95% of replicates were positive according to the Clinical and Laboratory Standards Institute guidelines. The limit of blank (LOB) was defined as the highest number of IS*6110* copies found in 12 blank samples (Armbruster and Pry, 2008).

### 2.7 ddPCR targeting IS*6110* data analysis

The QX200 Droplet Reader (Bio-Rad, USA) was used to read and count the fluorescent positive and negative droplets. Then, the data were analyzed using the QuantaSoft™ Analysis Pro software (version 1.0.596) (Bio-Rad, USA). Data from samples with 12,000 – 16,000 droplets were used for concentration calculations. Samples with a low number of droplets (< 10,000) were excluded from the analysis. According with the results for LOB, those samples with fewer than two positive droplets were considered “MTC-negative”, in contrast, samples were considered as “MTC-positive” when more than two droplets were found (Whale et al., 2020). Thus, the cut-off value of ddPCR-IS*6110* assay was set at 3 positive droplets in 20 μL reaction mixture.

### 2.8 Statistical analysis

The diagnostic performance of ddPCR targeting IS*6110* was evaluated for the detection of MTC in microbiological culture and fresh LN tissue samples. The diagnostic accuracy was compared with microbiological culture as the refence standard, considered as an imperfect reference technique for bTB diagnosis (Corner and Trajstman, 1988; Courcoul et al., 2014; Pucken et al., 2017). The adjusted sensitivity and specificity, false positive rate, false negative rate, positive likelihood ratio (PLR), and negative likelihood ratio (NLR) were calculated using Epidat 3.1 software. The PLR and NLR were interpreted according to the criteria published by Sackett et al. (2001), where a PLR > 10 or NLR < 0.1 indicates a technique of high diagnostic value that can discriminate between healthy and diseased animals, 5 < PLR ≤ 10 or NLR = 0.1-0.2 indicates a technique involving moderate changes in probability, 2 < PLR ≤ 5 or 0.2 < NLR ≤ 0.5 indicates a technique involving small changes in probability, and PLR ≤ 2 and NLR > 0.5 indicates rarely discernible changes. Finally, agreement between microbiological culture and ddPCR from culture, microbiological culture and ddPCR from fresh tissue, and real-time PCR and ddPCR both from fresh tissue was assessed using Cohen’s kappa coefficient (κ): κ = 0 indicated no agreement; 0.01 ≤ κ ≤ 0.20, slight agreement; 0.21 ≤ κ ≤ 0.40, fair agreement; 0.41 ≤ κ ≤ 0.60, moderate agreement; 0.61 ≤ κ ≤ 0.80, substantial agreement; and, 0.81 ≤ κ ≤ 1.00, almost perfect agreement (McHugh, 2012) (WinEpi software 2.0, Faculty of Veterinary Medicine, University of Zaragoza, Spain).

## 3 Results

### 3.1 Optimization of the ddPCR assay targeting IS*6110* for DNA isolated from microbiological culture and homogenized fresh tissue LNs

**Figure 1:**
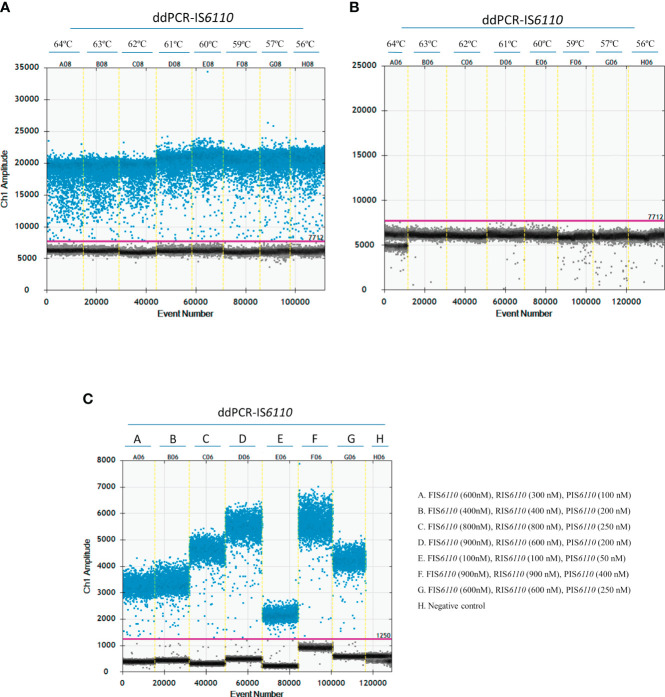
Optimization of the ddPCR-IS*6110* assay using *Mycobacterium tuberculosis* complex (MTC) DNA isolated from LN tissue samples. The gradient of annealing temperatures (ranging from 64°C to 56°C) for a positive sample **(A)** and non-template control (NTC) **(B)** were plotted with positive (blue) and negative (grey) droplets. E08 plotted the optimal temperature of annealing and extension (60°C). **(C)** Descending concentrations of primers and probe. D06 plotted the optimal primers and probe concentration assay (F-IS*6110* (900nM), R-IS*6110* (600 nM), P-IS*6110* (200 nM).

In order to optimize the ddPCR-IS*6110* assay, the first step was to determine an optimal annealing temperature, considered as one of the most critical parameters. Thus, we tested a range of annealing temperatures ranging from 56°C to 64°C. A total of 50 ng of MTC DNA isolated from a selective microbiological bacterial culture, 900 nM of forward and reverse primers together with 500 nM of probe were used in the assay. No restriction digestion of the DNA samples was performed. A negative template control (NTC) containing sterile water instead of DNA was included. We were able to detect the IS*6110* specific region in all the assessed temperatures (**Figure 1A**), however, the annealing temperature of 60°C (E08) showed a higher amplitude between positive and negative droplets compared with other temperatures and resulted in less non-specific amplification (rain). No positive droplets were observed in NTC for any of the temperatures (**Figure 1B**). Therefore, an annealing temperature of 60°C (E08) (**Figure 1A**) was selected as ideal temperature for further experiments.

Next step was to determine the optimal primer and probe concentrations for ddPCR-IS*6110* assay. Thus, five different concentrations of forward primer (100, 400, 600, 800 and 900 nM), reverse primer (100, 300, 400, 600, 800 and 900 nM) and probe (50, 100, 200, 250 and 400 nM) were tested (**Figure 1C**). Similarly, as above mentioned, a total of 50 ng of MTC DNA isolated from a selective bacterial culture were used in the assay, with no restriction digestion of DNA samples and inclusion of a NTC with sterile water instead of DNA. **Figure 1C** showed that the overall fluorescence amplitude of positive droplets increased with primers and probe concentrations. On the other hand, a much better amplitude between positive and negative droplets was observed when we used a concentration of 900 nM for forward, 600 nM for reverse and 200 nM for probe (**Figure 1C**, D06). This set up of primers and probes were used for further experiments.

**Figure 2:**
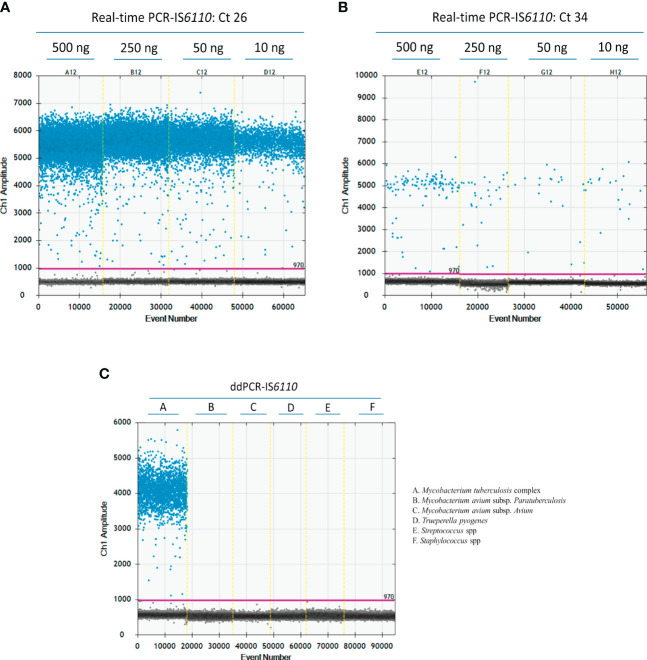
Optimization of the ddPCR-IS*6110* assay using *Mycobacterium tuberculosis* complex (MTC) DNA isolated from LN tissue samples. Descending concentrations of DNA isolated from MTC-positive samples with a Cq value of 26 **(A)** or 34 (500 ng, 250 ng, 50 ng, and 10 ng) in duplicate **(B)**. Analytical specificity of ddPCR-targeting IS*6110* evaluating the most common bacterial agents found in tuberculosis like lesion (*Mycobacterium avium* subsp. *paratuberculosis*, *Mycobacterium avium* subsp. *avium*, *Trueperella pyogenes*, *Streptococcus suis* and *Staphylococcus* spp.) **(C)**.

In the case of microbiological culture, we decided to use a sample concentration of 50 ng of DNA according to the concentration and volume available after DNA extraction. In the case of DNA isolated from fresh LN tissue, we were able to test the effect of sample quantity. A ddPCR assay was run using different concentrations of DNA from two different LNs that were IS*6110*-positive by real-time PCR with different Cq values (500 ng, 250ng, 50 ng and 10 ng) (**Figure 2A**, Cq = 26; and **Figure 2B**, Cq = 34). As illustrated in **Figure 2A**, a good separation between positive and negative droplets was observed at the four DNA concentrations (500 ng, 250ng, 50 ng and 10 ng) for DNA isolated from LN tissue samples. Because Poisson statistics test required enough negative droplets to be applied and calculate DNA concentration, we decided to use 50 ng (C12) of DNA isolated from tissue samples in further ddPCR assays. Also, this concentration could be a good fit to avoid cross-contamination in the case of samples with a high concentration of bacterial DNA.

### 3.2 Analytical specificity

The analytical specificity of ddPCR-targeting IS*6110* was tested against some of the most common microorganisms identified in TBL, such as *Mycobacterium avium* subsp. *paratuberculosis*, *Mycobacterium avium* subsp. *avium*, *Trueperella pyogenes*, *Streptococcus suis*, and *Staphylococcus* spp. (Cardoso-Toset et al., 2015). All these tests yielded negative results by ddPCR-IS*6110*, demonstrating the specificity of the primers and probe included in the study (**Figure 2C**).

### 3.3 Limit of blank and limit of detection

No positive droplets were found in 10 out of 12 blank samples (ddH_2_O instead of DNA sample), but one positive droplet per 20 μl reaction mix was detected in two of these blank samples. Accordingly, the limit of blank was set at 1 drop/20 μl reaction, and therefore, a sample was considered as “negative” when no more than two positive droplets were obtained.

To determine the LOD, 10-fold serial dilutions of *M. bovis* genomic DNA with known quantities ranging from 10^4^ to 10^0^ were used. The reactions were performed in triplicate for each dilution in three different assays. MTC IS*6110* sequences were detected in 100% of 10^1^ dilutions assayed; however, only 50% positivity was obtained at the level of 10^0^. Thus, LOD of this ddPCR targeting IS*6110* was set to 10^1^ copies per 20 µl reaction.

### 3.4 Comparison of confirmatory IS*6110* real time PCR and ddPCR from microbiological culture

As previously mentioned, microbiological culture positive samples need to be confirmed as MTC using real-time PCR (Thierry et al., 1990). Therefore, this part of the study compared both real-time PCR and ddPCR for the confirmation of culture positive samples. DNA was isolated from selective microbiological culture from 100 samples with gross TBL (N=19) or NVLs (N=81). Forty-nine out of 100 samples were tested as MTC-positive by real-time PCR (17 out of 19 with gross TBL) whereas 51 samples yielded a negative result and were classified as MTC-negative (**Table 2**). The Cq values for the real-time PCR targeting IS*6110* ranged from 20.00 to 36.25 (average = 29.35). No partial or complete inhibition were found in DNA isolated from microbiological culture. In the case of ddPCR targeting IS*6110*, forty-eight samples were found to be MTC-positive (19 out of 19 with gross TBL) and 52 MTC-negative (**Table 2**). There was an association between real time-PCR and ddPCR, with the higher number of positive droplets, which ranged from 13,402 to 4 droplets, coinciding with those samples with a lower Cq value.

**Table 2:** Evaluation of confirmatory real-time PCR and ddPCR targeting IS*6110* from microbiological culture DNA isolation according to the presence of gross tuberculosis-like lesions (TBL), histopathological TBL or no histopathological lesion (NHL).

		Real-time PCR-IS*6110* from microbiological culture		ddPCR-IS*6110* from microbiological culture	
		(+)	(-)	(+)	(-)
Gross TBL (n=19)*		17	2	19	0
**Histopathological TBL (n=57)**		34	23	37	20
**NHL (n=43)**		15	28	11	32

(+), positive; (-), negative.

*All the animals with gross TBL also presented histopathological TBL.

Analyzing the histopathological evaluation, 34 out of 49 samples positive to the microbiological culture by real-time PCR-IS*6110* were classified as TBL whereas 15 as NHL (**Table 2**). For the ddPCR-IS*6110*, histopathological TBL were found in 37 out of 48 ddPCR- IS*6110-*positive samples whereas 11 were classified as NHL (**Table 2**).

### 3.5 Comparison of IS*6110* real time PCR and ddPCR from fresh LN tissue samples

DNA was isolated from homogenized fresh LN tissue samples (N=100) with gross TBL (N=19) or NVL (N=81) and subjected to both real-time PCR and ddPCR. All the samples with gross TBL were found to be MTC-positive by both real-time PCR or ddPCR targeting IS*6110* (**Table 3**). For real-time PCR-IS*6110*, 53 out of 100 tested samples were detected as MTC-positive and 47 as negative. The Cq values ranged from 22.96 to 38.10 (average = 32.06). A partial inhibition of IAC was found in 6 out of 100 samples, with 2 out of 5 samples revealing a positive result after dilution 1:2 and re-evaluation. In the case of ddPCR, 55 samples were tested as MTC-positive and 45 as MTC-negative (**Table 3**). The number of positive droplets ranged from 12,618 to 3 droplets, with the highest number of positive droplets corresponding to those animals with pluribacillary lesions. As described before, an association was observed between real-time PCR and ddPCR, whereby a higher number of positive droplets corresponded to samples with higher Cq values.

**Table 3:** Evaluation of real-time PCR and ddPCR targeting IS*6110* from LNs DNA isolation according to the presence of gross tuberculosis-like lesions (TBL), histopathological TBL or no histopathological lesion (NHL).

		Real-time PCR-IS*6110* from LNs		ddPCR-IS*6110* from LNs	
		(+)	(-)	(+)	(-)
Gross TBL (n=19)*		19	0	19	0
**Histopathological TBL (n=57)**		40	17	41	16
**NHL (n=43)**		13	30	14	29

(+), positive; (-), negative.

*All the animals with gross TBL also presented histopathological TBL.

According to histopathological evaluation, 40 out of 53 positive samples to real-time PCR presented histopathological TBL whereas 13 did not present microscopic lesion (**Table 3**). Regarding ddPCR, 41 out of 55 positive samples were disclosed as positive to histopathology with 14 samples presenting NHL (**Table 3**).

### 3.6 Diagnostic performance of ddPCR-IS*6110* for the detection of MTC from microbiological culture and fresh LN tissue samples

In order to validate the ddPCR-IS*6110* from microbiological culture and fresh LN tissue samples, these assays were compared with the reference standard assay (selective microbiological culture confirmed by real-time PCR-IS*6110*). Assuming that this reference assay is considered an imperfect assay for performing MTC diagnosis (Corner et al., 2012; Courcoul et al., 2014; Pucken et al., 2017) validation was carried out using EPIDAT 3.1 software. The ddPCR-IS*6110* from microbiological culture detected 44 out of 49 reference standard positive samples [Se adjusted 90.76% (95% CI: 82.58-98.96%)], and 47 out of 51 reference standard negative samples resulted to be negative for ddPCR-IS*6110* [Sp adjusted = 100% (95% CI: 100%)]. Thus, an adjusted FNR of 9.23% (95% CI: 1.04-17.42%) and FPR of 0% (95% CI: 0%) were estimated (**Table 4**). The PLR value (PLR = ∞) implies a high diagnostic value for the positive results discriminating between MTC-infected and non-infected animals. In addition, the NLR value was 0.07 meaning that it is a technique of a high diagnostic value to discriminate between healthy and diseased animals (**Table 4**). Of note, all reference standard-negative but ddPCR-positive samples were also classified as MTC positive by histopathological evaluation or real-time-IS*6110* from fresh LNs tissue (**Table 5** summaries the discordant results between different diagnostic techniques).

**Table 4:** Diagnostic performance of droplet digital PCR (ddPCR) targeting IS*6110* from microbiological culture (A) and fresh lymph node (LN) tissue samples (B) in comparison with the established reference standard (selective microbiological culture confirmed by real-time PCR-IS*6110*) (N=100).

ddPCR- IS*6110* diagnostic accuracy (95 % CI)						
DNA source	Sensitivity	Specificity	FPR	FNR	PLR	NLR
(A)Microbiological culture	90.76 % (82.58-98.96 %)	100 % (100 %)	0 % (0 %)	9.23 % (1.04-17.23 %)	∞	0.07
(B) Fresh LNs tissue samples	94.80 % (88.52-100 %)	100 % (100 %)	0 % (0 %)	5.20 % (0-11.50 %)	∞	0.05

FPR, False positive ratio; FNR, False negative ratio; PLR, Positive likelihood ratio; NLR, Negative likelihood ratio; 95 % CI, 95 % confidence interval.

**Table 5:** Summary of discordant results between different diagnostic techniques.

ID	Histopathological lesion	Ziehl Neelsen	Reference standard protocol	Real-time PCR-IS*6110* from tissue	ddPCR-IS*6110* from microbiological culture	ddPCR-IS*6110* from tissue
11	No lesion	-	-	**+**	**+**	**+**
40	TB Granuloma	+ / Paucibacillary	-	**+**	**+**	**+**
49	TB Granuloma	+ / Paucibacillary	-	**+**	**+**	**+**
70	TB Granuloma	+ / Pluribacillary	-	**+**	-	**+**
73	MNGC	+ / Paucibacillary	-	-	-	**+**
77	TB Granuloma	-	-	**+**	-	**+**
101	TB Granuloma	-	-	**+**	-	**+**
110	MNGC	+ / Pluribacillary	-	**+**	-	**+**
161	TB Granuloma	+ / Paucibacillary	-	**+**	**+**	**+**

ID, identification; MNGC, Langhan’s type multinucleated giant cell; TB Granuloma, tuberculous granuloma; +, positive; -, negative.

In the case of ddPCR-IS*6110* carried out from fresh LN tissue samples, 47 out of 49 samples positive to the reference standard were found to be positive for ddPCR-IS*6110* [adjusted Se 94.80% (95% CI: 88.52-100%), and 42 out of 51 reference standard-negative samples were tested as ddPCR-IS*6110-*negative [adjusted Sp = 100% (95% CI: 100%)] (**Table 4**). According to these results, ddPCR-IS*6110* from fresh tissue presented an adjusted FNR of 5.20% (95% CI: 0-11.50%) and FPR of 0% (95 CI: 0%) (**Table 4**). Regardless of the true prevalence, ddPCR-IS*6110* had a high diagnostic utility to confirm and discard MTC infection (PLR = ∞ and NLR = 0.05) (**Table 4**). Noteworthy, 9 reference standard-negative but ddPCR-IS*6110-*positive samples were found also as positive by histopathological evaluation (8 out of 9) or real-time PCR-IS*6110* from fresh LN tissue (8 out of 9) (**Table 5** summaries the discordant results between different diagnostic techniques).

Finally, Cohen’s kappa coefficient (κ) showed an almost perfect agreement between the ddPCR-IS*6110* from culture and the reference standard (κ = 0.82) and a substantial agreement between the ddPCR-IS*6110* from fresh LN tissue samples and the reference standard (κ = 0.76).

## 4 Discussion

Selective microbiological culture followed by a confirmatory real-time PCR, despite being an imperfect assay with some limitations, is still considered the gold standard technique to confirm bTB infection (Taylor et al., 2007; Liebana et al., 2008; Courcoul et al., 2014). Thus, recovery rates for MTC culture oscillates between 30 and 95% (Hines et al., 2006; Corner et al., 2012; Courcoul et al., 2014; Yates et al., 2017;), depending on the preservation of samples until culture, the chemical decontamination process influencing the viability of MTC, the type of culture media chosen, and the extremely slow nature of MTC growth (Hines et al., 2006; Corner et al., 2012; Yates et al., 2017;). To address these challenges, the use of PCR for detecting MTC in animal tissue samples has been proposed as a rapid, accurate and sensitive alternative to conduct MTC confirmation (Lorente-Leal et al., 2019; Lorente-Leal et al., 2021; Sánchez-Carvajal et al., 2021; Vera-Salmoral et al., 2023). In this context, ddPCR, a third-generation PCR technology known for its ability to detect small amounts of nucleic acids with high precision and sensitivity, has been reported. Additionally, ddPCR has a high resistance to inhibitors due to sample partitioning (Baker, 2012; Pinheiro et al., 2012; Kuypers and Jerome, 2017). Therefore, ddPCR represents a promising alternative to other molecular diagnostic methods for instance real-time PCR (Baker, 2012; Pinheiro et al., 2012; Kuypers and Jerome, 2017). The present study aimed to develop and validate a ddPCR assay targeting IS*6110* to detect MTC in microbiological culture and fresh tissue samples with distinct TBL.

There are several performance parameters considered as key players in ddPCR including the concentration of primers and probe, the annealing temperature, or the quantity of the template (Whale et al., 2020). Optimization of these parameters is important to ensure the separation of positive and negative droplets and maximize the accuracy and sensitivity of the assay. Our results showed that the overall fluorescence amplitude of positive droplets increased with primers and probe concentrations. Thus, although we were able to detect the IS*6110* specific region in all the assessed setups, the annealing temperature of 60°C together with the primers and probe concentrations of 900 nM for forward, 600 nM for reverse and 200 nM for probe yielded a higher fluorescence amplitude between positive and negative droplets compared with other setups and resulted in less non-specific amplification. In the case of template concentration, no pre-digestion DNA steps were performed as the extraction protocol used in this study allows improving the detection of the MTC DNA with a minimal raining signal. In addition, a better diagnostic performance was demonstrated using this protocol for DNA isolation as we have previously reported for real-time PCR (Vera-Salmoral et al., 2023). Since the Poisson statistics test requires a sufficient number of negative droplets for accurate calculation of DNA concentration (Whale et al., 2020), we decided to proceed with a template DNA concentration of 50 ng from tissue sample. This concentration was chosen to ensure a suitable number of negative droplets for statistical analysis and to minimize the risk of cross-contamination in samples with high concentration of bacterial DNA.

Considering the multi-etiological nature of TBL (Cardoso-Toset et al., 2015a), we proceeded to assess the analytical specificity of ddPCR targeting IS*6110*. We tested several common microorganisms associated with TBL and observed that the primers and probe exhibited high specificity for MTC IS*6110*. The selection of an appropriate genetic target plays a key role for the accurate detection of MTC (Sevilla et al., 2015). Among the various targets available (Lorente-Leal et al., 2019; Wang et al., 2019), the insertion sequence IS*6110* is reported as one of the primary choices for diagnosing MTC (Sevilla et al., 2015). This genetic target not only enables differentiation between MTC and other bacteria, including non-tuberculous mycobacteria (NTM), but also offers the advantage of being a multicopy gene, ensuring sensitive and reliable detection of MTC (Charles et al., 2022). However, recent studies have identified the presence of an IS*6110*-like element in the genomes of certain NTM species, reporting a potential cross-reactivity between NTM and specific IS*6110* primer pairs or probes (Coros et al., 2008; Thacker et al., 2011; Michelet et al., 2018; Lorente-Leal et al., 2021). Nevertheless, the impact of these findings on the specificity of PCR-IS*6110* is expected to be minimal, as demonstrated in the following analysis.

ddPCR-IS*6110* demonstrated an adjusted Se of 90.77% (95% CI: 82.58 - 98.96%) and a Sp of 100% (95% CI: 100%) for the confirmation of MTC in selective bacterial culture when compared with the reference standard. The application of ddPCR in microbiological culture not only detected all samples with gross TBL but also increased the number of positive samples detected with NHL compared to real-time PCR-IS*6110*. However, it is noteworthy that five positive samples to the refence standard were classified as ddPCR-negative. It is possible that these cases represent false-negative results to ddPCR. One potential explanation could be the inhibition of the PCR reaction. Although the probability of this issue is low because ddPCR is known to be highly resistant to PCR inhibitors (Baker, 2012; Pinheiro et al., 2012; Kuypers and Jerome, 2017), ddPCR may remain to be susceptible to some inhibitors. To address the issue of uncertain results, including an IAC into the ddPCR assay can provide added reliability. By designing a duplex reaction, the IAC can be labelled in a separate channel during analysis using the Bio-Rad QX100/QX200™ Droplet Digital™ PCR system, which is capable of detecting duplex targets in two separate channels (FAM and VIC/HEX) when TaqMan hydrolysis probes are utilized. This approach allows for simultaneous detection of the target of interest, IS*6110*, and the IAC, providing an internal reference for assay performance and identifying any potential inhibition or technical issues during the analysis.

In the case of DNA isolated directly from fresh bovine LN tissue samples, ddPCR targeting IS*6110* proved to be a rapid and effective diagnostic assay when compared to traditional selective microbiological culture confirmed by real-time PCR. This ddPCR assay allowed us to detect all samples with gross TBL but also identified additional positive samples with microscopic lesions that were missed at the postmortem visual inspection. The ddPCR-IS*6110* assay showed an adjusted Se of 94.80% (95% CI: 88.52 - 100%) and a Sp of 100% (95% CI: 100%) demonstrating a significantly improved diagnostic performance and accuracy compared to the reference standard. Particularly, the ddPCR-IS*6110* assay disclosed as positive 9 samples negative to reference standard. Among these samples, 8 exhibited positive results in Ziehl-Neelsen staining and/or presented characteristic microscopic lesion. The remaining sample disclosed to be positive for both real-time and ddPCR-IS*6110* but negative to histopathology. These findings indicate the superior Se and Sp of ddPCR-IS*6110* directly from fresh tissue sample in detecting MTC compared to the reference standard. Furthermore, our findings reveal that ddPCR-IS*6110* exhibits significantly enhanced sensitivity and specificity when contrasted with real-time PCR-IS*6110* (Sánchez-Carvajal et al., 2021).

Although there have been no previous studies evaluating the diagnostic performance of ddPCR for MTC in animal samples, our research group conducted a preliminary approach using formalin-fixed paraffin-embedded (FFPE) tissue samples (Larenas-Muñoz et al., 2022). Our findings are consistent when compared to other studies conducted on human clinical samples (Song et al., 2018; Cho et al., 2020), reporting the rapid detection of MTC DNA. Furthermore, ddPCR offers advantages for MTC diagnostics across several sample types, including whole blood from patients with pulmonary and extrapulmonary TB lesion (Yang et al., 2017), culture isolates (Nyaruaba et al., 2020) or FFPE samples (Larenas-Muñoz et al., 2022). Additionally, our results demonstrated higher adjusted Se and Sp considering previous real-time PCR studies (Costa et al., 2013; Courcoul et al., 2014; Cardoso-Toset et al., 2015b; Lorente-Leal et al., 2019; Wang et al., 2019).

ddPCR technology offers several advantages over real-time PCR, making it an ideal technique for the detection of MTC, particularly in cases with a low-copy-number of the target (Kuypers and Jerome, 2017). Pathogens belonging to MTC are characterized by a paucibacillar pattern, which together the early detection of infected animals with NVL and low mycobacterial load would be beyond the LOD of traditional assays (Lorente-Leal et al., 2019; Sánchez-Carvajal et al., 2021). Due to sample partitioning, one notable advantage is the ddPCR ability to overcome the limitations caused by sample inhibitors which are commonly challenged in MTC samples (Dingle et al., 2013; Yang et al., 2014; Kuypers and Jerome, 2017). Additionally, ddPCR is less affected by poor amplification efficiency, further contributing to its robust performance in MTC detection (Kuypers and Jerome, 2017). Overall, these findings highlight the potential of ddPCR-IS*6110* as a valuable tool for accurate and sensitive MTC diagnosis which would be susceptible to be included in bTB routine confirmation procedure.

Nonetheless, this study has some limitations that also need to be addressed. Firstly, the number of samples might have been larger in order to increase the robustness of the results providing a more comprehensive evaluation. Also, the absence of a ring trial, which would involve multiple laboratories and diverse epidemiological scenarios, limits the external validation and applicability of the findings to broader contexts. Moreover, it is important to highlight some drawbacks of ddPCR system over other molecular techniques. In general, ddPCR is more time-consuming than real-time PCR. The chances of contamination are higher, the implementation of this assay also demands a higher level of technical expertise and specialized training for personnel involved in the procedure. In contrast, the cost per reaction in ddPCR is more cost-effective than other standard molecular methods, excluding the initial investment required to acquire the necessary equipment. Therefore, further work on the re-validation of the present protocol should be performed in the future.

The present study describes a complete protocol including sample pre-processing, DNA purification and ddPCR analysis. According to our results, ddPCR-IS*6110* demonstrated to be a rapid, highly sensitive and specific diagnostic tool as alternative to microbiological culture to confirm MTC infection shortening turnaround time for decision makers to be promptly informed. Comparing with real-time PCR, ddPCR has proved to be a potential first-choice molecular assay to detect MTC directly in fresh bovine tissue samples with increased Se and Sp. Therefore, ddPCR-IS*6110* approach has the potential to be included in bTB surveillance and control programs.

## Data availability statement

The original contributions presented in the study are included in the article/supplementary material. Further inquiries can be directed to the corresponding authors.

## Ethics statement

Ethical approval was not required for the study involving animals in accordance with the local legislation and institutional requirements because No purpose killing of animals was performed for this study, so no ethical or farmer’s consent approval was required.

## Author contributions

JS-C: Conceptualization, Investigation, Methodology, Validation, Writing – original draft. EV-S: Data curation, Formal Analysis, Investigation, Methodology, Writing – review & editing. BH: Data curation, Formal analysis, Software, Validation, Writing – review & editing. ÁG-R: Data curation, Methodology, Writing – review & editing. IR-T: Investigation, Methodology, Writing – review & editing. FL-M: Formal analysis, Investigation, Methodology, Writing – review & editing. IL: Funding acquisition, Project administration, Resources, Writing – review & editing. LC: Funding acquisition, Resources, Supervision, Writing – review & editing. JG-L: Conceptualization, Funding acquisition, Methodology, Validation, Writing – review & editing.
